# *Cystidicola farionis*, a Swim Bladder Parasite of European Smelt: Characterization of the Nematode Trehalose Strategy

**DOI:** 10.3390/ijerph19116430

**Published:** 2022-05-25

**Authors:** Małgorzata Dmitryjuk, Magdalena Szczotko, Katarzyna Kubiak, Janina Dziekońska-Rynko, Joanna Cichocka, Piotr Hliwa, Katarzyna Mierzejewska

**Affiliations:** 1 Department of Biochemistry, Faculty of Biology and Biotechnology, University of Warmia and Mazury, 10-719 Olsztyn, Poland; magdalena.szczotko@uwm.edu.pl; 2 Department of Medical Biology, School of Public Health, Collegium Medicum, University of Warmia and Mazury, 10-561 Olsztyn, Poland; katarzyna.kubiak@uwm.edu.pl; 3 Department of Zoology, Faculty of Biology and Biotechnology, University of Warmia and Mazury, 10-719 Olsztyn, Poland; jdr@uwm.edu.pl; 4 XI High School, 10-444 Olsztyn, Poland; jcichocka@cyfroweszkoly.pl; 5 Department of Ichthyology and Aquaculture, Faculty of Animal Bioengineering, University of Warmia and Mazury, 10-719 Olsztyn, Poland; phliwa@uwm.edu.pl

**Keywords:** parasite, Nematoda, *Cystidicola farionis*, *Osmerus eperlanus*, European smelt, trehalose, trehalose-6-phosphate synthase, TPS, trehalose-6-phosphate phosphatase, TPP

## Abstract

The molecular identification of *Cystidicola farionis* (a swim bladder nematode of European smelt from the Vistula Lagoon in Poland) was performed. Their prevalence level was determined, and changes in the trehalose synthesis pathway in larvae and adult nematodes were demonstrated. The trehalose level was almost four times higher in adult nematodes than in larvae. In contrast, the activity of both enzymes (trehalose 6-phosphate synthase, TPS and trehalose 6-phosphate phosphatase, TPP) involved in the synthesis of trehalose was higher in larvae than in adults under optimal conditions. The optimum pH for TPS isolated from larvae and adults was pH 7.0. The optimum pH for TPP from larvae and adults was pH 7.0 and pH 8.0, respectively. The optimal temperature was 20 °C, and Mg^2+^ ions were an activator for trehalose-synthetizing enzymes from both sources. Enzymes isolated from adult nematodes were less susceptible to divalent ion chelator and inorganic phosphate than larval enzymes. The dynamic transformation of trehalose in the nematode developing inside the swim bladder of the smelt appears to be an important metabolic pathway in the nematode survival strategy. These studies are aimed at a better understanding of the issue of the metabolic adaptation of parasites, which, in the future, may indirectly contribute to the elimination of the parasite from aquacultures, which will impact public health.

## 1. Introduction

The *Cystidicola farionis* Fischer, 1798 (Habronematoidea: Cystidicolidae) is a nematode parasitizing the swim bladder of fish mainly from the Salmonidae and Osmeridae families in Europe, Asia, and North America [1,2]. *Cystidicola farionis,* together with *Cystidicola stigmatura*, found only in North America, are considered the main species among 21 of the genus *Cystidicola*. The major features differentiating these species are the structure of fully developed eggs. In *C. farionis*, they are provided with long filaments, whereas *C. stigmatura* have lateral floats and a specific mouth shape [2,3]. The development cycle of nematodes of the genus *Cystidicola* is complex. The intermediate hosts are Amphipoda, including *Gammarus fasciatus*, *Hyalella azteca,* and *Pontoporeia affinis* [1,4]. In crustaceans, the nematodes reach the invasive third-stage larvae (L3). The final hosts of nematodes belong to open-bladder fish, which is why they are exposed to this parasite throughout their lives. The larvae penetrate the pneumatic channel and settle in the physostomous swim bladder, where they undergo the final molting and reach sexual maturity [5]. Fish infection with this parasite depends on many factors, including the age of the fish, the way and place of feeding, and the season of the year. Salmonidae above five years of age, mainly foraging in the littoral zone (the amphipod’s home), are the most vulnerable to acquiring this nematode. Since younger fish mainly feed in the profundal and pelagic zones, where these crustaceans do not occur, the intensity of infection of young fish is much lower [6,7,8].

The long life of the parasite–as well as the high intensity of the invasion, due to the host’s lack of reinvasion resistance–causes anemia and inflammation of the swim bladder (even leading to complete destruction of its walls). This parasite may also cause difficulties in filling the swim bladder, and disturb its hydrostatic and respiratory functions [2,9,10]. Cusack and Cone [11] emphasized the role of *C. farionis* as a vector of many bacterial diseases that additionally contribute to acute inflammation of the swim bladder wall. Parasites in the host’s body, in addition to action mechanical excreted toxins, are commonly referred to as excretion–secretion products. These products contain, among others, numerous hydrolases. Their higher activity in autumn tests is associated with the better condition of the parasite’s body, and thus its greater pathogenicity to the host, which may result in higher mortality of fish infected with this parasite in autumn [12].

There are few papers on the metabolism of *C. farionis* in the available literature. One study focused on the activity of selected hydrolases in excretion–secretion products and extracts from larvae and adults of *C. farionis* [12]. Another study researched the activity of carbohydrate catabolism enzymes in this nematode [13]. The purpose of the current study was to understand the synthesis pathway of trehalose, an important disaccharide for nematodes. Trehalose (α-D-glucopyranosyl-α-D- glucopyranoside) is a disaccharide with several key functions in nematodes: a protective substance in tissues for embryos, larvae, and adults during water deficit and cold stress, a source of energy and glucose storage, and a factor to facilitate egg larvae hatching. In most eukaryotic organisms, including nematodes, trehalose synthesis involves trehalose-6-phosphate synthase (TPS, EC 2.4.1.15) and trehalose-6-phosphate phosphatase (TPP, EC 3.1.3.12). The former (TPS) catalyzes the transfer of a glucose molecule from glucose uridine diphosphate (UDPG) to glucose 6-phosphate (G6P) to form the unstable trehalose 6-phosphate intermediate (T6P). The second enzyme (TPP) participates in the intermediate dephosphorylation to form the trehalose and inorganic phosphate molecule [14,15].

Therefore, this study determined the level of stored trehalose and the activity of enzymes participating in the trehalose synthesis pathway in larvae and adult forms of *C. farionis*, pre-determined the properties of enzymes involved in the synthesis of trehalose, and examined the effect of pH, temperature, and effector compounds on the activity of both enzymes involved in the synthesis of disaccharide. This study significantly contributes to understanding metabolic adaptations to the parasitic lifestyle of the *C. farionis* nematode.

## 2. Materials and Methods

### 2.1. Study Area and Sampling

**Figure 1:**
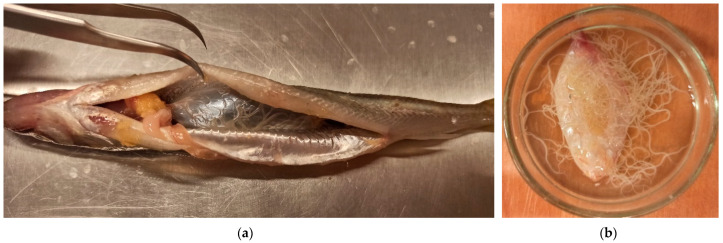
*Cystidicola farionis* parasitizing the swim bladder of European smelt *Osmerus eperlanus*; (**a**) Smelt dissection with swim bladder exposed; (**b**) Swim bladder with nematodes.

European smelt *Osmerus eperlanus* (caught with commercial fishing gear in the Vistula Lagoon [Poland; 54°27′00′ N, 19°45′00′ E] in spring 2021) were sampled. The fish were weighed and measured with accuracy up to 0.1 g and 0.1 mm, respectively, and were then dissected and analyzed for nematode presence in a swim bladder (Figure 1a). A total of 37 freshly sacrificed fish (26 females and 11 males) with a mean weight of 37.3 ± 6.8 g and a mean total length of 185.7 ± 11.8 mm were analyzed.

### 2.2. Nematode Identification and Preparation of Material for Research Trials

Nematodes isolated from all swim bladders were cleansed of host tissue debris by washing in 0.65% NaCl, and were then morphologically identified at the species level according to Grabda-Kazubska and Okulewicz [4]. Systematic features were observed under the microscope (NIKON ECLIPSE E200 LED equipped with a NIKON DSFi1 camera; NIKON Corporation, Tokyo, Japan). Larvae (L3 and L4; 899 specimens with lengths from 6 to 18 mm, with a pooled weight of 497.2 mg), as well as 375 juvenile and mature *C. farionis* (lengths from 19 to 31 mm, with a pooled weight of 1003.4 mg), constituted both research groups (larvae and adults). The material of a particular test group was divided equally into three parts and frozen at −80 °C until further procedures. The test extracts were prepared by homogenizing the sample weights with 0.65% NaCl in a ratio of 1:10 *w/v* using an Omni TH-02 homogenizer (5000–35,000 rpm; Omni International, Kennesaw, GA, USA). Homogenates were centrifuged at 2000× *g* for 10 min at 4 °C. The supernatant fraction was a complete extract for the determination of protein and trehalose content, activity of enzymes, and the determination of the biochemical properties of trehalose-6-phosphate synthase and phosphatase.

### 2.3. Molecular Identification of Cystidicola Farionis

In order to confirm the morphological identification of the parasites, six individuals (three larvae and three adults) were conserved in 70% ethanol and stored at 4 °C until DNA extraction. Extraction of DNA from whole single individuals of nematodes was carried out by the Sherlock AX universal kit (A&A Biotechnology, Gdynia, Poland) according to the manufacturer’s instructions. The nuclear large subunit rRNA gene region (28S rRNA) was amplified using the following primers (Nem28SF 5′-AGCGGAGGAAAAGAAACTAA-3′, Nem28SR 5′-TCGGAAGGAACCAGCTACTA-3′) and PCR conditions as described in Nadler et al. [16]. PCR products (~1000 bp) were purified using the CleanUp purification kit (A&A Biotechnology, Gdynia, Poland) according to the manufacturer’s protocol, and sequenced bi-directionally (Macrogen Europe, Amsterdam, the Netherlands). The obtained nucleotide sequences were edited in BioEdit v. 7.2 software (https://bioedit.software.informer.com, accessed on 10 February 2022) and compared with data registered in the GenBank database (http://www.ncbi.nih.gov/Genbank/index.html, accessed on 15 February 2022) using the BLAST-NCBI program (http://www.ncbi.nlm.nih.gov/BLAST/, accessed on 12 February 2022). Consensus partial sequences of the 28S rDNA of *C. farionis* were deposited in the GenBank database and registered under the accession numbers OM691415-OM691420. Obtained sequences and the most similar chosen reference sequences from GenBank were used in phylogenetic analysis. The phylogram was constructed using the Maximum Likelihood method based on the Kimura 2-parameter model. The topology of the phylogenetic tree was evaluated using the bootstrap method with 1000 replicates. Phylogenetic analysis was conducted using MEGA X software (https://www.megasoftware.net, accessed on 16 February 2022).

### 2.4. Determination of the Initial Properties of Enzymes—Optimum pH and Temperature

TPS and TPP activity in larvae and adults of *C. farionis* was initially demonstrated using modified methods described by Giaever et al. [17] and Kaasen et al. [18], respectively. To determine TPS activity, a mixture of 250 μL 0.1 M acid-ammonia buffer (pH range from 3.0 to 9.0), 50 μL 12.5 mM MgCl_2_, 50 μL 10 mM G6P, 50 μL 5 mM UDPG, and 100 μL full enzyme extract was used. After mixing, the respective controls were incubated at 100 °C for 5 min, and the test samples were incubated from 5–60 °C for 30 min and then at 100 °C for 5 min to complete the reaction. After cooling the samples, the obtained T6P was degraded by the addition of 100 μL solution of alkaline phosphatase (1 U) in a 0.1 M phosphate buffer (pH 8.0) at 37 °C for 30 min. The reaction mixture for determining TPP activity contained 300 μL 0.1 M acid-ammonia buffer (pH range from 6.0 to 9.0), 50 μL 12.5 mM MgCl_2_, 50 μL 2 mM T6P, and 100 μL extract full enzyme. After mixing, the respective control samples were incubated at 100 °C for 5 min; the tested samples were first incubated for 30 min in the temperature range of 5–60 °C and then boiled for 5 min. The final product of the reaction of both enzymes was determined according to Dmitryjuk et al. [19].

### 2.5. The Effect of Chemical Effectors on the Activity of Enzymes in the Trehalose Synthesis

In order to determine the effect of potential activators and inhibitors of reactions carried out by TPS and TPP, solutions of 1, 2, 5, 10, 50, and 100 mM MgCl_2,_ EDTA, and KH_2_PO_4_ were used. First, assays containing buffered enzyme protein and effectors were pre-incubated for 15 min at room temperature. The enzymatic reaction was then commenced by adding appropriate substrates. TPS samples contained 250 μL of optimal pH buffer (pH 7.0 for larvae and adults), 50 μL of an effector in an appropriate concentration, 50 μL of 10 mM G6P, 50 μL of 5 mM UDPG, and 100 μL of full enzyme extract. After mixing, the respective controls were incubated at 100 °C for 5 min, and the test samples were incubated at 20 °C for 30 min and then at 100 °C for 5 min to complete the reaction. In the next step, after cooling the samples, the obtained T6P was degraded by adding 100 μL of alkaline phosphatase solution according to the procedure described above. TPP samples contained 300 μL of optimal pH buffer (pH 7.0 for larvae and pH 8.0 for adults), 50 μL of an effector in an appropriate concentration, 50 μL of 2 mM T6P, and 100 μL of complete enzyme extract. After mixing, the respective controls were incubated at 100 °C for 5 min, and the test samples were incubated at 20 °C for 30 min and then at 100 °C for 5 min to complete the reaction. The trehalose obtained in both reactions was determined by the HPLC, as before. The value of trehalose obtained in the tested samples was reduced by the amount of trehalose found in the relevant control samples.

### 2.6. Determination of Trehalose Content and Demonstration of Optimal TPS and TPP Activity

Trehalose content was determined by high-performance liquid chromatography (HPLC) according to the procedure described by Dmitryjuk et al. [19]. To this end, the samples were prepared by boiling for 5 min and adding two volumes of ethanol and centrifugation. The resulting supernatants were then dried at 50 °C, dissolved in acetonitrile/deionized water (3: 2, *v/v*), and filtered using Micro-Spin Filter tubes (Alltech Associates, CA, USA). Next, 20 μL of each test was applied to an injector on a Shimadzu SCL-10A system equipped with a RID 10 A refractometer detector (Kyoto, Japan) and a high-performance carbohydrate cartridge column (4.6 × 250 mm; Waters, the Netherlands). Samples were eluted from the column at 35 °C using a mixture of acetonitrile: degassed deionized water (75/25%, 1 mL/min). The trehalose content in the samples was analyzed using Chromax 2005 software (POL-LAB; Warsaw, Poland).

A mixture for determining TPS and TPP activities was prepared as above and contained an addition of 50 μL 10 mM MgCl_2_. After mixing, the respective controls and test samples were prepared as above. Reactions were conducted at optimal pH (TPS: pH 7.0 for larvae and adults; TPP: pH 7.0 and pH 8.0 for larvae and adults, respectively) and temperature (20 °C). The final product of the reaction of both enzymes, i.e., trehalose, was determined as above [19].

TPS and TPP activity is expressed in units (U) based on mg of protein measured by the Bradford method [20] using BSA as the standard. One unit indicates the amount of trehalose (nM) obtained during 1 min reaction in optimal conditions. The trehalose content is expressed in mg based on 1 g of fresh tissue. Both study groups (larvae and adult nematodes) were pooled into three independent groups, and trials for analysis were prepared in triplicate (*n* = 9). The results (Mean ± SD) were analyzed by one-way analysis of variance (ANOVA) in the Statistica 12 program (StatSoft Inc., Tulsa, OK, USA). Tukey’s honestly significant difference test was used to show statistically significant differences between means. *p*-values of *p* < 0.01 were considered statistically significant.

## 3. Results

### 3.1. Prevalence and Intensity of Infestation in Fish

**Figure 2:**
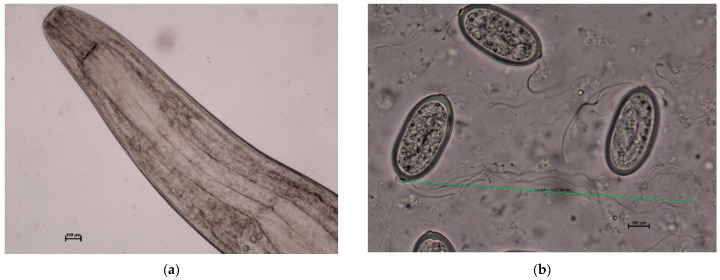
*Cystidicola farionis*; (**a**) anterior region of an adult female (scale bar 100 μm); (**b**) Eggs removed from the uterus provided with characteristic filaments (scale bar 100 μm).

Out of 37 fish, *C. farionis* nematodes (Figure 2a) were identified in 20 swim bladders (20/37, 54.05%) (Figure 1a). There were 12 infected females (12/26, 46.2%) and eight infected male smelt (8/11, 72.7%). Up to 223 individual nematodes were found in the swim bladders of the fish (Figure 1b). The uteri of mature female nematodes were filled with eggs measuring 453.1 ± 2.5 μm in length and 214 ± 13.6 μm in width (area: 81,059.1 ± 3749.7 μm^2^) with characteristic filaments (2–8 in each bunch) of medium length 1204.1 ± 222.2 μm (Figure 2b).

### 3.2. Molecular Identification of Cystidicola Farionis

**Figure 3:**
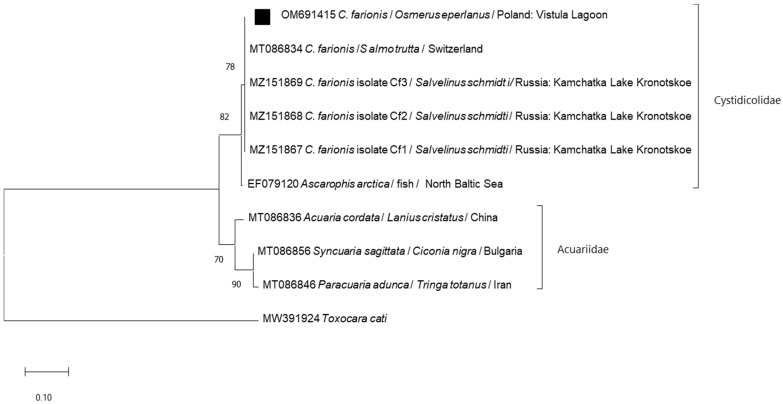
Molecular relationships of the sequence of the nuclear large subunit rRNA gene region (28S rRNA) of *Cystidicola farionis* identified in the study with other species from Cystidicolidae and Acuariidae. The phylogenetic tree was constructed using the neighbor-joining method and the Kimura 2-parameter as a distance method. Numbers at the tree nodes indicate the percent of bootstrap value from 1000 replicates. The tree is drawn to scale, with branch lengths measured in the number of base substitutions per site. *Toxocara cati* was used as an outgroup. The analyses were conducted in MEGA X. The sequence obtained in this study was labelled with black symbols.

Analysis of the nucleotide sequences of six PCR positive samples confirmed 100% identity to the fragment of 28S rRNA gene of those previously detected in *C. farionis* from other fish, including *Salmo trutta* from Switzerland (MT086834) and *Salvelinius schmidti* from Russia (MZ151867-69) (Figure 3). The obtained sequences (OM691415-OM691420) were identical to each other, which does not indicate intraspecific variability of nucleotides of the 28S rRNA gene from *C. farionis* parasitizing the swim bladders of European smelt.

### 3.3. Activity of Trehalose Synthesis Enzymes

#### 3.3.1. Determination of the Optimal pH of TPS and TPP

**Figure 4:**
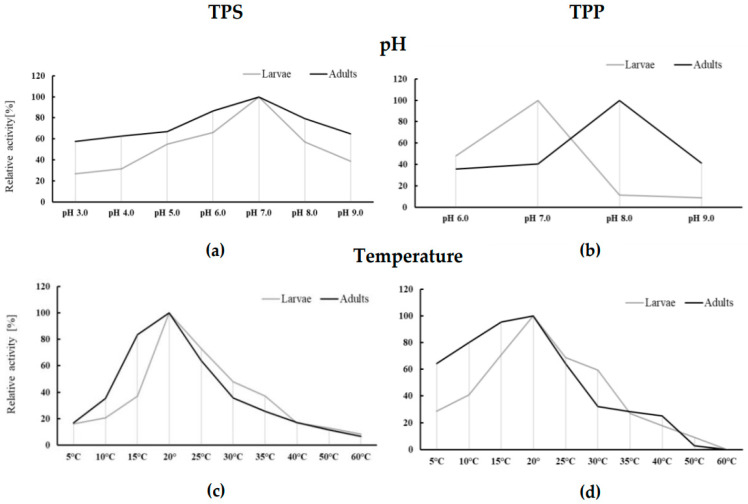
Dependence of activity of trehalose-6-phosphate synthase (TPS) and treha-lose-6-phosphate phosphatase (TPP) from larval and adult *Cystidicola farionis* on pH (**a**,**b**) and temperature (**c**,**d**); (**a**) The maximum activities of TPS at pH 7.0 for larvae (712.75 ± 210.86 U/mg) and adults (329.74 ± 60.40 U/mg] were taken as 100%; (**b**) The maximum activities of TPP at pH 7.0 for larvae (1033.20 ± 84.18 U/mg) and pH 8.0 for adults (859.78 ± 113.87 U/mg) were taken as 100%; (**c**) The maximum activities of TPS at temperature 20 °C for larvae (2320.62 ± 347.97 U/mg) and for adults (666.08 ± 80.50 U/mg) were taken as 100%; (**d**) The maximum activities of TPP at temperature 20 °C for larvae (1337.82 ± 101.31 U/mg) and for adults (860.43 ± 69.78 U/mg) were taken as 100%; (**a**–**d**) Mean ± SD; *n* = 9.

TPS from both sources showed activity over the entire tested pH range of 3.0–9.0. The optimum pH for TPS isolated from larvae (L3 and L4) and adults was pH 7.0. The enzyme isolated from adult parasites was more resistant to changes in pH of the reaction environment than the enzyme from larvae. In acidic conditions (pH 3.0), it revealed more than half of the maximum activity, and in an extremely basic environment (pH 9.0), it showed over 60% of the maximum activity (Figure 4a). TPPs isolated from larvae and adults showed activity in the tested pH range 6.0–9.0. The optimal pH of enzyme activity from larvae and adults was pH 7.0 and pH 8.0, respectively. The enzyme isolated from larvae was more resistant to acidification of the reaction environment than its alkalization. At pH 6.0, the enzyme from this source revealed almost half the maximum activity, while at pH 8.0–9.0, it showed only trace activity. TPP isolated from adult parasites (like TPS) was less sensitive to changes in pH of the reaction medium than larval phosphatase (Figure 4b).

#### 3.3.2. Determining the Optimal TPS and TPP Operating Temperature

The optimal temperature for trehalose synthesis pathway enzymes from both sources was 20 °C (Figure 4c,d). In the case of TPS, the increase in the reaction temperature up to 35 °C caused a loss of enzyme activity from larvae by about 66% and from adult nematodes by up to 75%. At 60 °C, the enzyme from both parasite stages revealed trace activity (Figure 4c). TPP from both sources was an enzyme more sensitive to the rise in reaction temperature than TPS. At 50 °C, the enzyme activity from larvae and adult nematodes was very low, and at 60 °C, the enzymes from both origins were inactive (Figure 4d).

#### 3.3.3. The Effect of Chemical Effectors on the Activity of Enzymes in the Trehalose Synthesis Pathway

**Figure 5:**
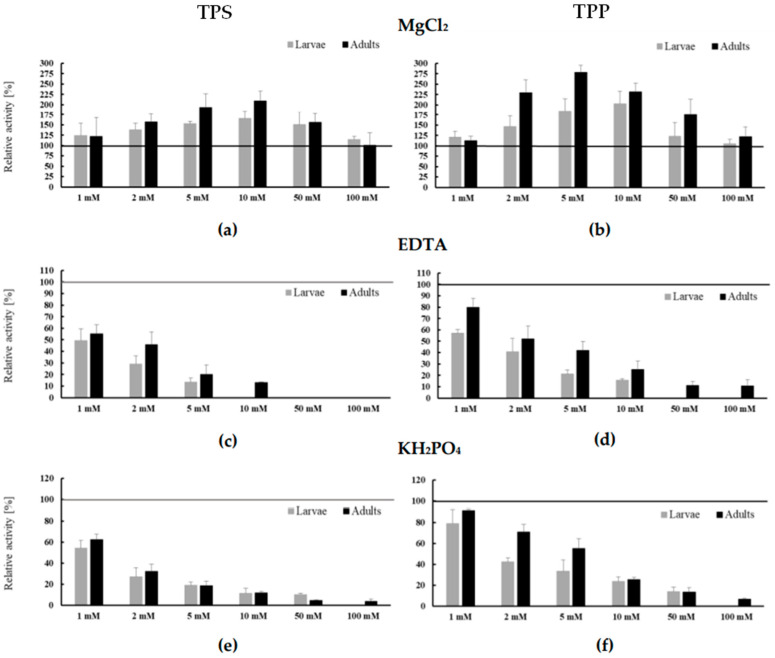
Influence of different concentrations of effectors on the activity of trehalose-6-phosphate synthase (TPS) and trehalose-6-phosphate phosphatase (TPP); (**a**) The activities of TPS control samples without effector—MgCl_2_ (Mg^2+^) (459.19 ± 84.21 U/mg for larvae and 206.7 ± 29.79 U/mg for adults) were taken as 100%; (**b**) The activities of TPP control samples without effector—MgCl_2_ (Mg^2+^) (635.98 ± 59.28 U/mg for larvae and 240.38 ± 5.6 U/mg for adults) were taken as 100%; (**c**) The activities of TPS control samples without effector—EDTA (815.08 ± 180.04 U/mg for larvae and 600.01 ± 60.5 U/mg for adults) were taken as 100%; (**d**) The activities of TPP control samples without effector—EDTA (549.58 ± 117.67 U/mg for larvae and 347.69 ± 105.38 U/mg for adults) were taken as 100%; (**e**) The activities of TPS control samples without effector—KH_2_PO_4_ (Pi) (595.47 ± 62.9 U/mg for larvae and 251.53 ± 29.94 U/mg for adults) were taken as 100%; (**f**) The activities of TPP control samples without effector—KH_2_PO_4_ (Pi) (704.94 ± 58.52 U/mg for larvae and 310.84 ± 18.13 U/mg for adults) were taken as 100%; (**a**–**f**) Mean ± SD; *n* = 9.

As a result of the conducted analyses, it was shown that MgCl_2_ in all tested concentrations was an activator of both synthase and trehalose-6-phosphate phosphatase in larvae and adults of *C. farionis*. Mg^2+^ ions most effectively activated TPS in adults and nematode larvae at a concentration of 10 mM (approximately 109% and 65%, respectively). Higher magnesium chloride concentrations did not activate the enzyme significantly. In the adult parasite, the TPS activity in the presence of 100 mM MgCl_2_ was only 100.66% and 114.9% for larvae (Figure 5a). In turn, 5 and 10 mM magnesium chloride concentrations most effectively increased the TPP activity. An increase of 85% and 102% in TPP activity was observed in larvae at the above chloride concentrations, respectively. In adult parasites, it was an increase of as much as 178% and 132% for these MgCl_2_ concentrations. In the presence of higher concentrations of magnesium ions, a significant weakening of the enzyme-activating effect was observed. In larvae extracts in the presence of 50 and 100 mM chloride solutions, an increase in TPP activity of 23% and 5% was observed. In adult nematodes, this increase was by 75% and 21.5%, respectively (Figure 5b). Test concentrations of EDTA and KH_2_PO_4_ solutions had an inhibitory effect on both enzymes of the trehalose synthesis pathway. Along with the increase in inhibitor concentration, a gradual decrease in activity was observed for TPS and TPP from both nematode groups (Figure 5c–f). A 1 mM EDTA solution reduced the activity of TPS from both sources by approximately half. For the larval enzyme, the use of 10 mM and higher chelator concentrations in the reaction mixture completely inhibited TPS activity. In adult nematodes, the same effect was observed with 50–100 mM EDTA concentrations (Figure 5c). TPP showed a lower EDTA inhibitory effect than TPS. This particularly concerned the enzyme isolated from adult nematodes. The application of 1 mM EDTA reduced TPP activity by only about 20%, and 2–5 mM by nearly half. In the presence of 50–100 mM inhibitor solutions, very low enzyme activity was noted (Figure 5d).

The enzyme isolated from larvae was more susceptible to the inhibitory effects of EDTA. TPP from this source in the presence of 1 mM chelator showed 57.1% of maximum activity. At higher concentrations, the inhibitory effect of the factor increased. When using a 50 mM EDTA solution, TPP from larvae was no longer active (Figure 5d). In the case of the second inhibitor (KH_2_PO_4_), enzymes of the trehalose synthesis pathway isolated from adult nematodes turned out to be more resistant to its action, similar to the case of EDTA. Both enzymes isolated from larvae (TPS and TPP) were blocked by a 100 mM phosphate solution. Enzymes isolated from adult *C. farionis* also maintained low activity in the presence of the highest concentrations of the inhibitor (Figure 5d,f).

#### 3.3.4. Trehalose Content and TPS and TPP Activity under Optimal Conditions

Adult *C. farionis* accumulated significantly more trehalose (nearly four times higher) than their larvae, and the differences between the means of both groups were highly statistically significant (*p* < 0.01). The level of activity of enzymes involved in the synthesis of trehalose was not correlated with the content of the disaccharide tested. The activity of both enzymes was significantly higher in larvae than in adults. The differences between the activity of both enzymes in larvae and adult nematodes were statistically significant (*p* < 0.01; Table 1).

## 4. Discussion

Due to its important role in the food web and a life cycle covering different environments, *O. eperlanus* is a suitable model for ecological and parasitological studies [21]. European smelt fishing has not only culinary but also social, economic, and cultural dimensions [22]. For this reason, the spread of parasitic infections due to fish movement is a major problem for fish living in artificial and natural water reservoirs, as well as for public health [23]. There are no reports on the pathogenicity of *C. farionis* to other animals or to humans [24]. However, a negative relationship between *C. farionis* prevalence and palmitoleic acid concentrations in wild lake whitefish (*Coregonus clupeaformis*) has been found, suggesting a decrease in the nutritional status of infested fish [25]. These studies are intended to lead to a better understanding of the issue of the metabolic adaptation of parasites to survive in the swim bladder of *O. eperlanus*, which in the future may indirectly contribute to the elimination of the parasite from aquacultures.

The morphological identification of *C. farionis* nematodes in European smelt in the Vistula Lagoon was confirmed based on the amplification of regions within the nuclear large subunit ribosomal DNA (28S) containing two highly variable domains–D2 and D3 [26]. Sequence analysis of those rDNA regions indicated that the studied nematodes (both analyzed larvae and adult nematodes) belong to the *C. farionis* species. In studied specimens of *C. farionis,* a lack of variation within the fragment of the 28S rRNA gene was noted. A similar lack of variability in the same part of the 28S rRNA gene was also demonstrated by Kashinskaya et al. [27] in *C. farionis* from nosed charr (*Salvelinus malma*) in Lake Kronotskoe in Kamchatka, Russia. Moreover, sequences from examined nematodes from European smelt in the Vistula Lagoon form a homogeneous group together with those obtained from parasites from *S. schmidti* in Russia and *S. trutta* in Switzerland.

It is important to study the metabolism of the *C. farionis* parasite in order to learn about its survival strategy in the host because the prevalence of *C. farionis* in harvested catches in the Vistula Lagoon is high and ranges from 25.9% to 75% [24]. A high intensity of infection can cause fish anemia and inflammation of the swim bladder, worsening its hydrostatic and respiratory function. A large population of nematodes can lead to the destruction of the swim bladder walls and may increase mortality rates of fish [2,7,9]. Many nematodes also require sugars as obligatory substrates for energy metabolism. Sugar is mainly stored in nematodes in the form of glycogen and partly as disaccharide trehalose which, in addition to glucose storage, performs a number of physiological functions important for the survival of nematodes [15]. Żółtowska et al. [13] showed a relatively low level of glycogen in *C. farionis* tissues compared to other nematodes. The level of polysaccharides was 2.5% and around 5% for larvae and adult parasites. These results were in line with previous observations by Von Brand [28] that nematodes feeding on blood and host tissues are in a more stable position when it comes to food availability than intestinal parasites or free-living nematodes. Therefore, the level of sugar they accumulate does not have to be high. In the present study, a relatively high level of trehalose was observed in adult nematodes (about 1.43% of the weight of nematodes) and a significantly lower level of this disaccharide was observed in the larval forms of *C. farionis* (0.37%). Żółtowska et al. [13] received much lower values for the same nematode using the enzymatic method to determine the level of trehalose (0.2% and 0.3% for larvae and adults, respectively). In both experiments, more trehalose was observed in adult nematodes. This can be explained by the increased energy demand of mature nematodes associated with the reproductive process. Similarly, the parasitic nematode *Anisakis simplex* had a successive increase in trehalose in subsequent larval stages. The content of the disaccharide in L4 larvae was almost five times higher than in L3 larvae [29].

TPS in nematodes synthesizes the intermediate trehalose-6-phosphate, the accumulation of which may have lethal effects due to its toxicity [30]. TPP, however, belongs to the superfamily of HAD-like hydrolases and has strong and specific phosphatase activity in relation to the trehalose-6-phosphate substrate. In closely related nematodes, the presence of conserved active TPP sites and their absence in hosts has been observed. This makes the trehalose synthesis pathway a promising site for new antiparasitic drugs [31]. Moreover, in *C. farionis*, trehalose synthesis appears to be an important pathway in the survival strategy of these nematodes. Nematodes accumulate a significant amount of trehalose, and the differences in the intensity of transformation in larvae and adult nematodes are significant. The presented studies showed, for the first time, the activity of trehalose synthesis pathway characteristic of other nematodes, involving trehalose-6-phosphate synthase and phosphatase in both larvae and adults of *C. farionis*. However, as mentioned above, lower trehalose levels were observed in the parasite larvae, and the enzyme activity of the trehalose synthesis pathway was nearly 1/3 higher at this stage compared to adult nematodes. A similar relationship was observed in the case of another nematode *Contracaecum rudolphii*, in which increased activity of the trehalose synthesis pathway enzymes was observed in juvenile parasites compared to adults [32]. This is probably related to the intense growth of nematodes during this period and the need to accumulate easily metabolized sugar for further stages of parasite development. At this stage, the metabolism of juvenile nematode forms is probably geared to disaccharide synthesis. This can be confirmed by the results obtained by Żółtowska et al. [13]; the authors observed significantly lower activity of trehalase (trehalose-hydrolyzing enzyme) in nematode larvae, as well as trehalose phosphorylase (an enzyme that breaks down trehalose by phosphorolysis) than in adult parasites. Trehalose synthesis pathway enzymes (TPS and TPP) isolated from *C. farionis*, showed the highest activity in the range of pH 7.0–8.0. TPS isolated from both stages was most active at neutral pH 7.0, similar to *A. simplex* L3 synthase [33]. During nematode development, a change in the optimal pH of TPP was observed from neutral pH 7.0 in larvae to slightly alkaline pH 8.0 in adult parasites. Similar results were obtained for the enzymes of the trehalose synthesis pathway from *A. simplex* L3 larvae, where both enzymes showed an optimum pH 7.0. In addition, phosphatase isolated from the muscles of the adult nematode *Ascaris suum* had an optimum pH 7.0 [34]. This is in contrast to the purified TPS from porcine roundworm muscles, which showed the highest activity in an acidic environment with a pH of 3.8–4.2 [14]. Trehalose metabolism enzymes isolated from *C. farionis* had a relatively low optimum temperature (20 °C for both stages studied). This is perhaps related to the adaptation to life in a host preferring a relatively low water temperature. Because *C. farionis* nematodes–parasitizing cold-blooded fish in the swim bladder–get there as L3, undergo subsequent molting, and reach sexual maturity, the metabolism of both larvae and mature individuals takes place under varying temperature conditions. This remains in opposition to the test results for nematodes parasitizing warm-blooded animals. In the case of TPS and TPP from porcine roundworm muscles, the optimal operating temperature was a temperature close to the body temperature of the nematode host, i.e., 35 °C [14,34]. The discussed enzymes from the *A. simplex* L3 stage, whose paratenic hosts are marine fish (herring, cod, mackerel) and the ultimate hosts of warm-blooded marine mammals, also had a much higher temperature optimum (40 °C and 45 °C for TPP and TPS, respectively) [33]. *Anisakis* L3 larvae are most often found in the body cavity in the pyloric processes of the fish digestive system, and thus their living environment has a more stable temperature than the living environment of *C. farionis* larvae and adults.

In addition to determining the optimal pH and temperature of trehalose synthesis enzymes in larvae and adult nematodes, it has been shown that Mg^2+^ ions are the activator of the pathway, as in other nematodes *A. suum*, *A. simplex*, and *Brugia malayi* [14,33,34,35] or representatives of other systematic groups *Pleurotus tuoliensis* [36], *Pseudomonas putida,* and *Thermus thermophilus* [37,38]. It has also been shown that the divalent EDTA chelator and inorganic phosphate are inhibitors of the trehalose synthesis pathway and exert a stronger effect on *C. farionis* larvae. In the case of EDTA, a similar effect was observed by Dmitryjuk et al. [14,34] and Kushwaha et al. [35] in the nematodes *A. suum* and *B. malayi*, respectively. In contrast, in the *A. simplex* third larvae stage, ethylenediaminetetraacetic acid lowered TPP activity at low concentrations and activated the trehalose synthetizing enzymes at 20 mM [33]. An increase was also observed in the inhibitory effect on the activity of enzymes involved in the synthesis of trehalose, with an increase in the concentration of P_i_ in the reaction environment. A similar relationship was observed by Cai et al. [39] for purified TPS from *Metarhizium anisopliae*. Other researchers showed that inorganic phosphate at physiological doses may be a non-competitive inhibitor for TPS1 from *Saccharomyces cerevisiae* [40,41]. Alternatively, Bell et al. [42] showed that P_i_ activates TPS1 from yeast, and Fiol and Salerno [43] confirmed that 20 mM inorganic phosphate did not affect the purified TPS/TPP complex from *Euglena gracilis*.

Summing up the most important achievements of the presented research, it should be emphasized that *C. farionis* has low variability based on parts of the 28S gene and does not display intraspecific variability of nucleotides of this gene. Moreover, for the first time, the path of trehalose synthesis in the nematode *C. farionis* (parasitizing the swim bladder) was determined. The optimal pH of TPS and TPP enzymes is a neutral environment for both larvae and adults. The optimal temperature for trehalose synthesis pathway enzymes is relatively low (20 °C). Mg^2+^ ions in concentrations of 5–10 mM most effectively activate both synthases and trehalose-6-phosphate phosphatase in larvae and adults of *C. farionis*. The divalent EDTA ionizer and inorganic phosphate inhibit nematode trehalose synthesis pathway enzymes, with TPS and TPP isolated from adult nematodes being more resistant to the inhibitory effects of both effectors than larval enzymes.

## 5. Conclusions

The species identification of the parasitic nematode from the swim bladder of European smelt (a fish caught in the Vistula Lagoon), i.e., *C. farionis*, was confirmed by morphological and molecular analyses, and the prevalence of nematode was shown to be high (54%). The presented study, for the first time, revealed the activity of a trehalose synthesis pathway typical for other nematodes, involving trehalose-6-phosphate synthase and phosphatase in both larvae and adults of *C. farionis*. Dynamic transformation of trehalose in the nematodes developing in the swim bladder of the smelt seems to be an important metabolic pathway in the survival strategy of this species.

## Figures and Tables

**Table 1:** Content of trehalose and optimal activities of trehalose-synthetizing enzymes from *Cystidicola farionis* (Mean ± SD).

Development Stage	Trehalose Content (mg/g of Fresh Tissue)	TPS Activity (U/mg of Proteins)	TPP Activity (U/mg of Proteins)
Larvae	3.75 ± 0.5 *	591.16 ± 107.97 *	729.2 ± 58.1 *
Adults	14.27 ± 0.68	355.37 ± 42.15	579.22 ± 53.91

* *p* < 0.01 for larvae vs. adults.

## Data Availability

The data presented in this study are contained within the article.
